# Pathogenic Characterization of European Genotype Porcine Reproductive and Respiratory Syndrome Virus Recently Isolated in Mainland China

**DOI:** 10.2174/1874357901711010083

**Published:** 2017-06-30

**Authors:** Sun Ming, Ma Yongying, Liu Bohua, Lu Huiying, Deng Xiaoyu, Liu Qiaorong, Qiao Mingming, Chen Xi, Yang Xinyan, Chen Xizhao

**Affiliations:** Beijing Anheal Laboratories Co., Ltd, Beijing 100094, China

**Keywords:** Pathogenicity, Newly emerged Chinese type I PRRSV isolate

## Abstract

**Background:**

Porcine reproductive and respiratory syndrome virus (PRRSV) is an important pathogen in pig that causes tremendous economic loss in the global swine industry. PRRSV is divided into the European and North American genotypes, with virulence ranging from apathogenic-moderately virulent to highly pathogenic. The emergence of new highly virulent type 1 strains and coexistence of the two genotypes complicate the differential diagnosis, disease prevention, and control of PRRSV. Although the emergence of a novel type 1 PRRSV strain in mainland China was first confirmed in 2011, there is no information available concerning the pathogenesis of this strain.

**Objectives:**

We sought to determine the pathogenesis of a newly emerged Chinese type 1 PRRSV strain HLJB1.

**Methods:**

Pigs were infected with HLJB1 and characterized using serological and histopathological tests.

**Results:**

HLJB1 infection induced transient chemosis, reddened conjunctiva, skin cyanosis, mild transient pyrexia, dyspnea, and tachypnea between 7 and 13 days post-infection. Gross pneumonic lesions were characterized by multifocal, tan-mottled areas. Lymph nodes and spleen were enlarged. Characteristic microscopic lesions consisted of pulmonary consolidation and alveolar septal thickening with red blood cell infiltration, depletion of splenic lymphocytes, and hyperplasia and activation of macrophage. No pigs infected with HLJB1 died during the experiment.

**Conclusion:**

Our findings indicate that Chinese type I PRRSV strain HLJB1 caused classic PRRSV-specific lesions. As it caused lower viremia in pigs compared with other classic type 1 isolates, HLJB1 is less virulent than other type I strains.

## INTRODUCTION

Porcine reproductive and respiratory syndrome virus (PRRSV) is a small, enveloped, positive-stranded RNA virus that belongs to the order Nidovirales, family Arteriviridae, and genus Arterivirus [1]. PRRSV is the causative agent of porcine reproductive and respiratory syndrome (PRRS). PRRS has become one of the most significant and economically important infectious diseases affecting swine worldwide, characterized by reproductive failure in sows and respiratory problems in piglets and growing pigs [2]. PRRSV shows a high degree of genetic variation [3, 4] and some antigenic heterogeneity [5, 6]. Based on genetic and antigenic characteristics, PRRSV is divided into two distinct genotypes: the European genotype (type I), with the prototype strain Lelystad virus representing viruses predominately originating from Europe; and the North American genotype (type II), with the prototype strain VR-2332 representing viruses predominately originating from North America. Both genotypes share approximately 55–70% nucleotide identity [7]. There is increasing diversity among strains of the two genotypes, which has been attributed to the high error rate inherent in PRRSV replication and recombination between strains [8]. According to recent reports on PRRSV classification, type I is divided into three subtypes: a pan-European subtype 1 and East European subtypes 2 and 3 [9]. Historically, type 1 was restricted to Europe and type 2 to North America; however, coexistence of the two genotypes has been identified in Europe, North America, and Asia, complicating the differential diagnosis, disease prevention, and control of PRRSV [10, 11]. These genotypes cause similar clinical signs but exhibit significantly different virulence potentials, ranging from apathogenic-moderately virulent to highly pathogenic. Type 2 PRRSV strains induce more severe respiratory disease than type 1 PRRSV strains [12-14].

During the last decade, new highly virulent strains have emerged in Eastern Europe, China, Vietnam, and South Korea, causing more severe clinical signs [15-17]. An initial outbreak of PRRS in mainland China occurred at the end of 1995 and has since become widespread. The current infection rate of Chinese swine herds has risen to 90%, with PRRS now one of the most significant problems for Chinese swine production [18]. Research on PRRSV in China has primarily focused on North American PRRSV isolates, particularly highly pathogenic PRRSV isolates in recent years [19-21]. In contrast, only a few Chinese type 1 PRRSV sequences have been submitted to the GenBank database with no corresponding research. Therefore, there is a lack of information about the virulence of Chinese type 1 PRRSV strains.

HLJB1 is a newly emerged type 1 PRRSV strain recently isolated from pig in mainland China, and its complete genomic sequence has been determined (GenBank accession no. KT224385.1). Phylogenetic analysis of type 1 PRRSV strains based on the nucleotide sequences of complete ORF5 genes indicated that HLJB1 belongs to a separate phylogenetic branch from other known type 1 PRRSV strains. The aim of this study was to characterize the pathogenicity of recently isolated Chinese type 1 PRRSV strain HLJB1 in pigs after experimental infection.

## MATERIALS AND METHODS

### Viruses

HLJB1 (passage 5) was propagated in pulmonary alveolar macrophages at a titer of 1×10^5^ TCID_50_/ml in Dulbecco’s modified Eagle’s medium supplemented with 5% fetal bovine serum, penicillin (100 units/ml), and streptomycin (100 ug/ml).

### Animals And Experimental Design

At 8 weeks of age, nine conventional pigs from a PRRSV-negative herd were purchased from a PRRSV-free commercial farm. All pigs were PRRSV-negative according to routine serology and real-time PCR (RT-PCR) analysis prior to delivery and on arrival. All pigs were individually housed throughout the experiment in an environmentally controlled building within pens over completely slatted floors. The pigs were randomly assigned to two groups and kept for 7 days to allow adaptation to the new conditions. The pigs in group 1 (n = 7) were inoculated oronasally with 10^5^ TCID_50_/pig HLJB1 in 2 ml of phosphate-buffered saline (PBS). The pigs in group 2 (n = 2) were inoculated oronasally with 2 ml of PBS and served as negative controls. Clinical signs (body temperature, respiratory disorders, and general signs such as appetite and behavior) were monitored daily in both groups starting from 6 days pre-inoculation until the day of death or euthanasia.

### Serological Tests

Blood samples from HLJB1-inoculated pigs were collected at 0, 7, 14, 21, and 30 days post-infection (dpi). Serum samples were tested using a commercially available PRRSV enzyme-linked immunosorbent assay (ELISA; HerdChek PRRS 2XR, IDEXX Inc., USA). Serum samples were considered positive for PRRSV antibody if the S/P ratio was greater than 0.4 according to the manufacturer’s instructions.

### Gross And Histopathological Tests

At 10, 15, and 21 dpi, three pigs were euthanized and lungs, tonsils, spleen, and inguinal lymph node samples were collected. Two samples from each location were collected. One sample was frozen and stored at -70°C, while the other was fixed in 4% formaldehyde solution and further processed for histopathology using hematoxylin and eosin staining.

### Quantification of PRRSV RNA

The QIAamp Viral RNA Mini kit (QIAGEN GmbH, D-40724 Hilden) was used to obtain viral RNA from serum samples. A 140-ul volume of serum was used for the extraction. RT-PCR for type 1 PRRSV was performed to quantify PRRSV genomic cDNA copy numbers, as previously described [22].

## RESULTS

### Clinical Signs

**Fig. (1):**
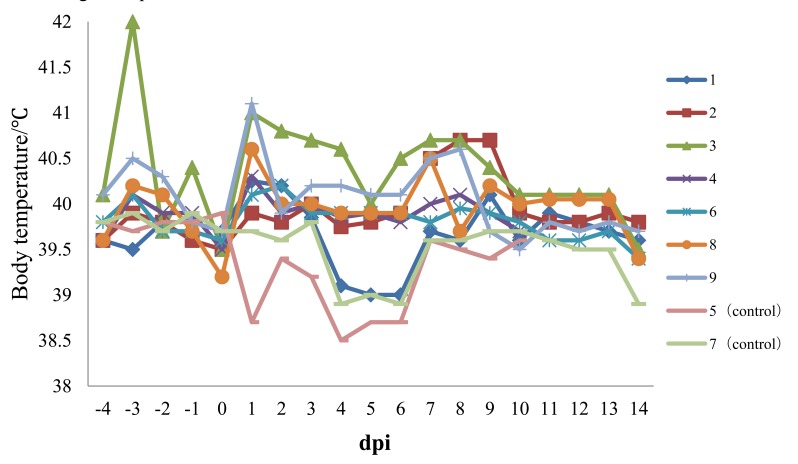
Body temperature of pigs following infection with HLJB1. Temperature ≥40°C was considered as fever.

Experimental infection of Chinese type 1 PRRSV strain HLJB1 in pig induced fever, transient chemosis, reddened conjunctiva, skin cyanosis, mild transient pyrexia, dyspnea, and tachypnea. At 2 dpi, six of seven pigs already had fever (body temperatures ≥40°C) that lasted for more than 3 days (Fig. **1**). Anorexia and depression were seen in one pig from 11 to 13 dpi. All seven pigs exhibited respiratory symptoms to varying degrees; wheezing was observed in one pig at 2 dpi, one at 4 dpi, four at 8 dpi, and one last 2 to 5 days. Ear skin cyanosis was observed in two pigs from 7 to 10 dpi. Two pigs showed chemosis and reddened conjunctiva from 8 to 15 dpi. Thereafter, body temperatures returned to normal, and all animals recovered and stayed healthy and alive until the end of the experiment. No pigs infected with HLJB1 died during the experiment.

### Serology of PRRSV

**Fig. (2):**
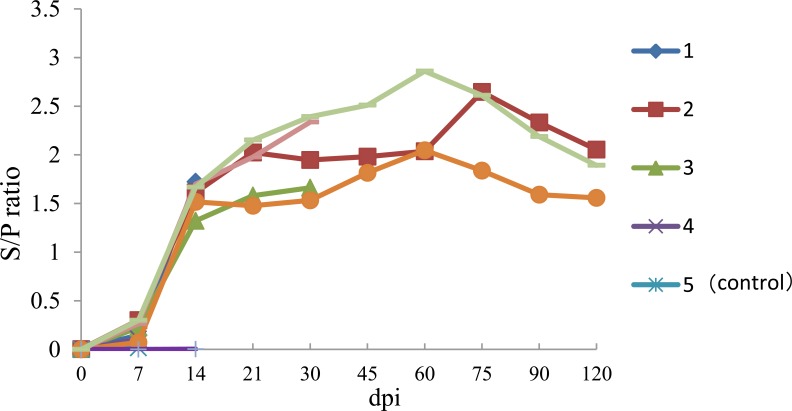
Body temperature of pigs following infection with HLJB1. Temperature ≥40°C was considered as fever.

Anti-PRRSV IgG antibodies were detected in infected pig as early as 7 dpi and all infected pigs were found to be seropositive by 10 dpi. Thereafter, all infected pigs remained seropositive for PRRSV (Fig. **2**). No anti-PRRSV IgG antibodies were detected in the serum of negative control pigs throughout the experiment.

## RSV-Specific Serum IgG

### Gross and histopathological tests

**Fig. (3):**
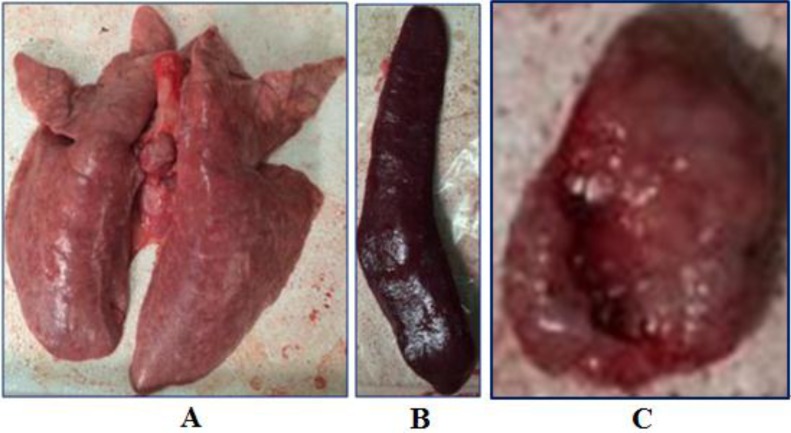
Body temperature of pigs following infection with HLJB1. Temperature ≥40°C was considered as fever.

**Fig. (4):**
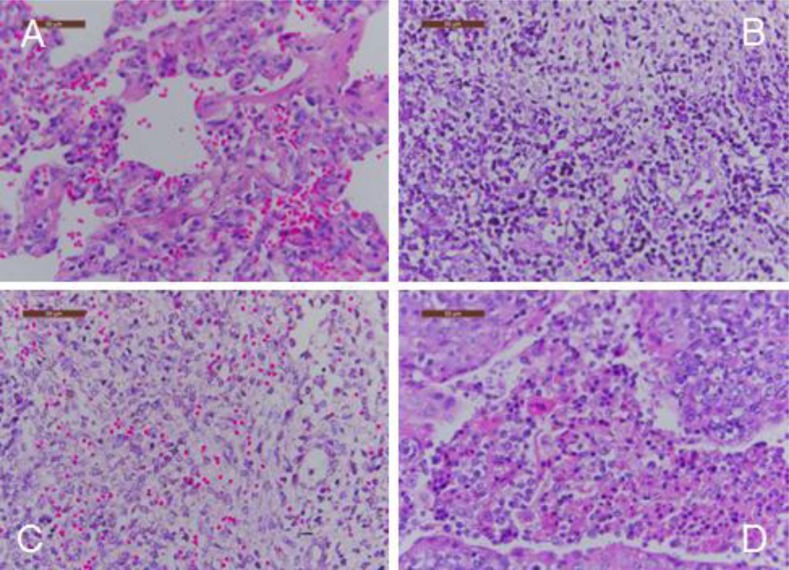
Body temperature of pigs following infection with HLJB1. Temperature ≥40°C was considered as fever.

Gross pneumonic lesions were characterized by multifocal, tan-mottled areas with irregular and indistinct borders (Fig. **3**). Lymph nodes were tan in color and enlarged. Spleens were enlarged and consolidated, with some containing infarcts. Characteristic microscopic lesions consisted of pulmonary consolidation and septal thickening with red blood cell infiltration, depletion of splenic lymphocytes, hyperplasia and macrophage activation, lymphocytic degeneration, necrosis, and red blood cell infiltration. Lymph nodes lesions included depletion of lymphocytes, and the cortex was infiltrated with a large number of red blood cells. Tonsils had necrotic foci (Fig. **4**).

### Viremia

**Fig. (5):**
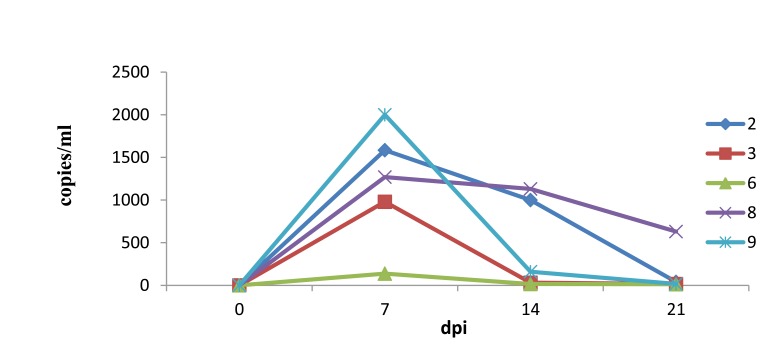
Viral load in the serum as determined by quantitative real-time PCR.

As there were a limited number of serum samples, the exact time viral titers were first detected in sera was not determined. However, the viral load in sera of HLJB1-infected pigs peaked at 7 dpi, and viremia in all pigs but one lasted until 21 dpi or longer (Fig. **5**).

## DISCUSSION

Lelystad virus is the prototype strain of type I PRRSV and represents viruses predominately originating in Europe. Experimental infection of Lelystad virus in pigs showed that the virus could induce mild transient pyrexia, dyspnea, tachypnea, and multifocal tan-mottled consolidation. Characteristic microscopic lung lesions consisted of type 2 pneumocyte hypertrophy and hyperplasia, necrotic debris and increased mixed inflammatory cells in alveolar spaces, alveolar septal infiltration with mononuclear cells, lymphadenopathy with follicular hypertrophy, hyperplasia, and necrosis [12]. In contrast, the newly emerged East European subtype 3 PRRSV strain Lena showed pathogenic severity in conventional pigs. High fever, anorexia, and depression were prominent signs in most pigs inoculated with Lena between 2 and 28 dpi. Fever (temperature range, 40–42.1°C and lasting for several weeks), prolonged viremia (lasting until 28 dpi), co-infection with bacterial pathogens, abnormally high viral load in sera (ranging from 10^4.0^ to 10^6.1^ TCID_50_/ml at 10 dpi and 10^4.3^ to 10^6.0^ TCID_50_/ml at 14 dpi.), high viral titers in tissues, and high mortality were prominent findings [17]. Additionally, Lyoo et al. recently described two field cases of type 1 PRRSV infection with unusually severe pathogenicity that occurred in South Korea [15]. Severe respiratory distress was seen in pigs of the grower-to-finish unit, with mortality rates ranging from 22% and 50% [15].

In the present study, the pathogenicity of a newly emerging type I PRRSV strain (HLJB1) in mainland China was evaluated in young pigs. Like other classic type 1 PRRSV isolates, the novel Chinese type 1 strain HLJB1 induced mild clinical signs such as pyrexia and dyspnea upon experimental infection of young pigs. Gross pneumonic lesions with enlarged lymph nodes and spleen were observed. Characteristic microscopic lesions consisted of pulmonary consolidation, depletion of splenic lymphocytes, hyperplasia and macrophage activation, lymphocytic degeneration, and necrosis. The clinical signs and pathological changes were all similar to that of type 1 PRRSV strains in previous reports. Nevertheless, HLJB1 caused lower viremia compared with other classic type 1 isolates, and viremia in most pigs infected with HLJB1 lasted only 2–3 weeks. As higher and prolonged viremia is one of the prominent characteristics of highly pathogenic type I PRRSV strains, the results in this study suggested that HLJB1 is less virulent than other type I strains. Despite lower viremia, HLJB1 induced a good immune response in infected pigs (Fig. **2**), suggesting the potential of type I PRRSV HLJB1 as a vaccine candidate.

## CONCLUSION

In conclusion, the newly emerging Chinese type I PRRSV isolate HLJB1 used in this study could induce classical PRRSV-specific lesions. As it caused lower viremia in pigs compared with other classic type 1 isolates, HLJB1 is less virulent than other type I strains.
